# Shock Absorption Behavior of Elastic Polymers for Sports Mouthguards: An In Vitro Comparison of Thermoplastic Forming and Additive Manufacturing

**DOI:** 10.3390/ma15082928

**Published:** 2022-04-17

**Authors:** Philipp Schewe, Ariadne Roehler, Sebastian Spintzyk, Fabian Huettig

**Affiliations:** 1 Department of Prosthodontics, Center for Dentistry, Oral Medicine and Maxillofacial Surgery, University Hospital Tuebingen, 72076 Tübingen, Germany; philipp.schewe@student.uni-tuebingen.de (P.S.); fabian.huettig@med.uni-tuebingen.de (F.H.); 2 Medical Materials Science and Technology, University Hospital Tuebingen, 72076 Tübingen, Germany; s.spintzyk@fh-kaernten.at; 3 ADMiRE Lab—Additive Manufacturing, Intelligent Robotics, Sensors and Engineering, School of Engineering and IT, Carinthia University of Applied Sciences, 9524 Villach, Austria

**Keywords:** intraoral splints, 3D printing, sports medicine, dentistry, trauma, rapid manufacturing, thermoforming

## Abstract

Background. There are several in vitro testing options to investigate the efficacy of sports mouthguards. None of these represent everyday situations, but the effects of simple laws of physics can be observed. This enables the comparison of conventional materials for mouthguards towards fabrications from additive manufacturing. Methods. A ball-drop experiment measured the maximum force and temporospatial distribution of a vertical impact on six material groups and a reference group (No-MG). Three conventional materials (ethylenvinylacetate) with 1, 2, and 3 layers were compared with additively manufactured (AM) specimens of comparable layering with a respective thickness of 4 mm, 5 mm, and 6.8 mm. Results. A maximum force of 8982.35 N ± 305.18 (No-MG) was maximum damped to 2470.60 N ± 87.00 (conventional 6.8 mm) compared with 5585.09 N ± 203.99 (AM 6.8 mm) Thereby, the ratio between shock absorption per millimeter was best for 4 mm thickness with means of 1722 N (conventional) and 624 N (AM). Conclusions. Polymer layers demonstrated a force reduction up to 71.68%. For now, additively processed resins of comparable hardness and layering are inferior to conventional fabrications.

## 1. Introduction

Mechanical impacts to the teeth and jaw can happen in a wide range of activities, especially combat sports, ball sports or sports with a high risk of falling such as skiing, skating, or biking [1,2,3]. Therefore, it is advisable to wear a sports mouthguard [4]. This measure should prevent the fracture or dislocation mainly of the upper anterior teeth, predominantly exposed in the lower face. The mechanical impact of a moving object is physically characterized by the momentum $\vec{p}$ and can be calculated considering the object mass *m* and the velocity $\vec{v}$:

(1) $$\vec{p}=m\cdot \vec{v}$$

Additionally, the momentum transfer $d\vec{p}$ from an object to a tooth is also defined by a force $\vec{F}$ and the transfer time dt:

(2) $$d\vec{p}=\vec{F}\cdot dt$$

So, the effectiveness of a mouthguard protecting the teeth from an impacting object is defined by two factors, the maximum force $\vec{F}$ and the temporal spreading of the force dt. The destructive shock absorption of a mouthguard is characterized by two properties: a damping of the maximum force and a temporospatial spreading of the impact momentum [5,6].

The gold standard in mouthguard manufacturing is the thermoplastic forming of thin ethylenvinylacetate (EVA) foils over a plaster model of the (mostly) upper jaw, also misleadingly called “deep drawing” technique [7]. The thermoforming procedure goes in hand with inconsistencies of thickness, and therewith a low reproducibility in thickness. In the worst case, the mouthguard is not fulfilling the intended protection in the field situation [8].

Generally, such EVA foils are delivered by manufactures as one, two, or even multi-layer blanks. This allows the application of industrial fabricated, combined degrees of shore hardness to adapt to the risks for individual field situations/sports [9]. More layers go in hand with a higher thickness and a reduced wearing comfort [10].

With the advent of digitalization in dental medicine emerged new materials, manufacturing methods and possibilities [11]. The so called “polyjet” additive manufacturing (AM) method applies a liquid of photopolymer to a platform in layers of 16 to 32 µm via a print head with linearly arranged nozzles. The polymer is directly polymerized by an ultraviolet (UV) light source implemented into the print head. This processing method allows the simultaneous application of various materials (i.e., different shore hardness) within one object. This implies that the UV-polymerized resin is rather plastic (flexible towards physical forces) and not thermoplastic (gaining more elasticity with rising temperature). The additive manufacturing of fully digital-designed mouthguards promises a fast, reproducible (esp. regarding thickness), and individualized solution [12,13]. Disadvantages of thermoplastic forming such as thinning [14] and delamination [15] could be overcome with AM. Even if such individual and reproducible additive layering is possible, the applied materials (AM polymers) have to prove their competence in shock absorption; measured against conventionally manufactured mouthguards (from thermoplastic polymers), first. Therefore, in vitro tests about their damping behavior are necessary when it comes to mouthguards.

Previous studies provided several experimental setups in order to measure either the absorption of impacting maximum forces or the temporospatial spreading of forces [16,17]. On the one hand, such testing set-ups can include sophisticated approaches, including the simulation of natural movement of the teeth within the jaw [18]. On the other hand, test set-ups can reduce the number of confounders and focus on the materials’ behavior only. Highly simplified test setups with a small number of influencing factors can ensure a better comparability of materials between different studies and working groups. Such an approach was published by Chowdhury et al. with a ball-drop test, observing the maximum impact force in an experimental setup as simple as possible [16].

An experimental in vitro study clarifies the damping behavior of polymers applied in conventionally layered mouthguards as well as that applied in additive manufacturing of comparable layer thicknesses. To the authors knowledge, it is the first investigation of AM-fabricated potential polymers for mouthguard application towards the damping behavior. This very behavior is an important in vitro result to know before testing novel materials for mouthguards in human application because in vivo medical device testing of mouthguards according to ISO 14155 would also include the exposition of the subjects to real-life impact and, therefore, the applied materials should be at least equal to the gold-standard treatment. In addition, other properties such as biocompatibility in the oral cavity for transient use have to be clarified to gain medical allowance.

The test design should overcome the incomparability due to complex experimental setup. Therefore, the study is purely to test the mechanical damping properties of different polymers and not their clinical suitability.

The null hypothesis of the study is that there are no statistically significant differences in damping behavior between the conventional and AM-fabricated group of the same thickness.

## 2. Materials and Methods

### 2.1. Test Setup

**Figure 1:**
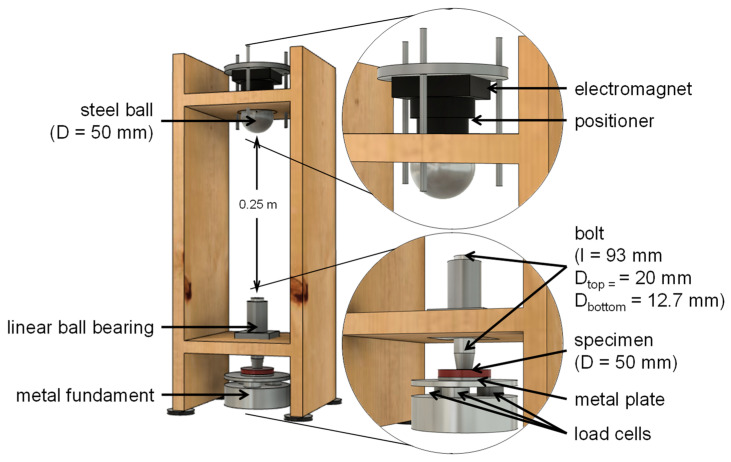
Schematic drawing of the test setup assembled in a wooden frame: the steel ball was released from the electromagnet to fall on the bolt (D_top_ = 20 mm) inserted in a linear bearing to transfer the load (D_bottom_ = 12.7 mm) to the specimen (D = 50 mm) laying on the plate borne by three load cells.

A ball-drop test—based on the descriptions of Chowdhury et al.—was constructed (cf. Figure 1) in order to evaluate the shock absorption of specimens in three thicknesses (4 mm, 5 mm, and 6.8 mm) made from conventional blanks for thermoplastic forming (conventional group; see Table 1) as well as from polymers for additive manufacturing in the polyjet technique (AM group, see Table 2). The kinetic energy was accomplished by a steel ball (524 g, D = 50 mm) falling vertically released from an electromagnet. The kinetic energy was transmitted by an interposed bolt lying on the polymer specimen which was placed on a metal plate. Underneath, the resulting kinetic energy was recorded by three identical load cells (KM26, ME-Messsysteme GmbH, Henningsdorf, Germany) placed in a metal fundament. A specialized software (GSVMulti 1.47’, ME-Messsysteme GmbH, Henningsdorf, Germany) recorded 16,000 load measurements per second from each cell.

### 2.2. Specimen Fabrication

In order to achieve different heights and hardnesses for the conventional specimens, two different manufacturers where chosen. The specimens, transparent and thermoplastic EVA sheets with different thicknesses, were vacuum molded on a plane gypsum plate of 70 mm in diameter after controlled heating according to manufacturers’ instructions in a thermoplastic former (ERKOFORM-RVE, 0.8 bar, Erkodent Erich Kopp GmbH, Pfalzgrafenweiler, Germany and BIOSTAR, 5.3 bar, SCHEU-DENTAL GmbH, Iserlohn, Germany). The specimens were cut with a radius of 25 mm from the center of the plate (D = 50 mm). The groups, the exact composition, and processing parameters of the test specimens are shown in Table 1.

The AM-group specimens were fabricated by the manufacturer from two polymers (mixture of a “rubberlike” product called Agilus30 and a rather “stiff” material called Vero) according to the thicknesses and shore hardness of the conventional EVA specimens. A mixture of the two mentioned polyjet polymers was necessary in order to achieve the desired shore hardnesses. Both materials are not yet medically approved polymers for prototyping. The delivering company Silconic (Lonsee, Germany) provided the solid specimens manufactured from an STL file with the cylinder design (50 mm diameter and varying heights) printed in polyjet procedure with an Objet260 Connex3 (Stratasys, Rechovot, Israel) according to the indicated layering in Table 2.

A total of seven specimens per group were fabricated. The thickness of each specimen was measured with a caliper (IP54, HELIOS-PREISSER GmbH, Gammertingen, Germany) on five random sites prior to the impact tests.

### 2.3. Conduction of the Test and Calculations

The ball-drop experiment was repeated five times for all seven specimens in each of the six groups (N = 35 measurements per group). Additionally, N = 5 force measurements were performed in absence of a specimen as a reference. These measurements were grouped as “No-MG”.

The force over time was recorded for the three load cells separately. The resulting force was calculated by adding up the raw data of the load cells. The maximum force was determined and the ball’s impact onto the polymer specimen was extracted. With the measured thickness, a thickness-dependent force [N/mm] was calculated. In addition, the relative shock absorption of the individual group averages compared with the reference group No-MG was calculated.

As in this experimental setup, the ball’s drop is accelerated by the earth’s gravitational acceleration constant $g=9.81\;\frac{m}{s^{2}},$ and the distance between the ball and the bolt is given by the height h=0.25 m; the velocity $\vec{v}$ can be calculated by:

(3) $$h=\frac{1}{2}\cdot g\cdot t^{2}\to t=\sqrt{\frac{2h}{g}}\;\mathrm{and}\;\vec{v}=g\cdot t$$

According to Newton’s third law of motion (action = reaction), the momentum of the entire system is maintained during a collision of two objects. The momentum sum of all moving objects before the collision $\vec{p}$ equals the momentum sum of all objects after the collision $\vec{p^{\prime }}$. Together with Equation (1), the following results for the two moving objects ball and bolt:

(4) $$\sum \vec{p}=\sum \vec{p^{\prime }}\;m_{\mathrm{ball}}\cdot \vec{v_{\mathrm{ball}}}+m_{\mathrm{bolt}}\cdot \vec{v_{\mathrm{bolt}}}=m_{\mathrm{ball}}\cdot \vec{v_{\mathrm{ball}}^{\prime }}+m_{\mathrm{bolt}}\cdot \vec{v_{\mathrm{bolt}}^{\prime }}$$

After the impact the ball is moving back upwards. For Equation (4), the direction of velocity has to be respected as mathematical signs (↓ ≜+ and ↑ ≜−). Assuming the momentum transfer $d\vec{p}$ corresponds to the total momentum, the velocities of the bolt before and after the impact are zero, and other momentums are negligible; this results in:

(5) $$d\vec{p}=m_{\mathrm{ball}}\cdot \vec{v_{\mathrm{ball}}}-(-m_{\mathrm{ball}}\cdot \vec{v_{\mathrm{ball}}^{\prime }})$$

The momentum transfer $d\vec{p}\;$ is calculatable from the recorded force over time data:

(6) $$d\vec{p}=\int_{t_{1}}^{t_{n}}\vec{F}\left(t\right)\cdot dt$$

All data were tested for normality with Shapiro–Wilk W test (alpha = 0.05) and for statistical significance with Kruskal–Wallis test (alpha = 0.05) in JMP 15 (SAS Institute, Cary, NC, USA).

## 3. Results

The initial thickness of the conventional specimens had a higher standard deviation (1L-Conv-4: ±91 µm, 2L-Conv-5: ±43 µm, 3L-Conv-6.8: ±132 µm) than the additively manufactured (1L-AM-4: ±4 µm, 2L-AM-5: ±3 µm, 3L-AM-6.8: ±5 µm) specimens.

**Figure 2:**
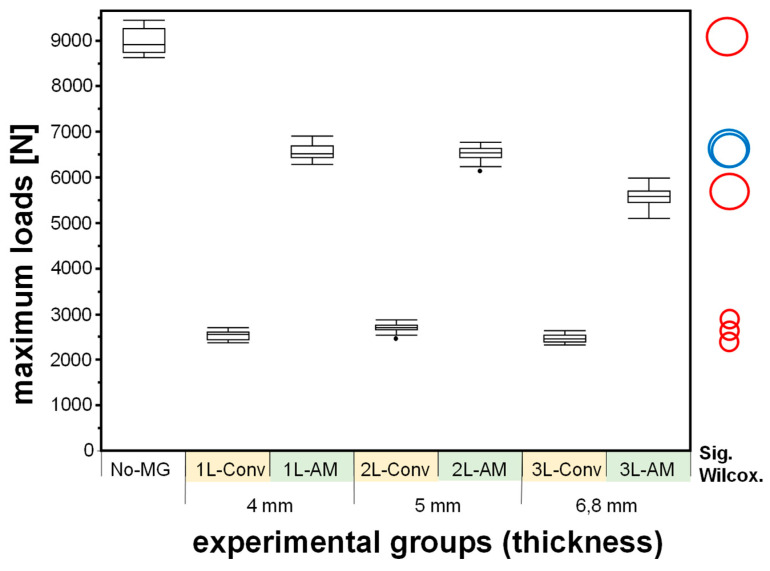
Boxplot diagram of the maximum loads (in N, y-axis) measured in the experimental groups (N = 35 and x-axis) and reference (No-MG, N = 5, and x-axis). The comparison circles plot on the right side indicates the confidence interval by the circles’ diameter and the statistical significance between two groups (Wilcoxon multiple comparison, *p* < 0.05) if the angle of intersection of the corresponding circles is fewer than 90° (red). An intersection angle of more than 90° indicates statistical insignificance (blue).

**Figure 3:**
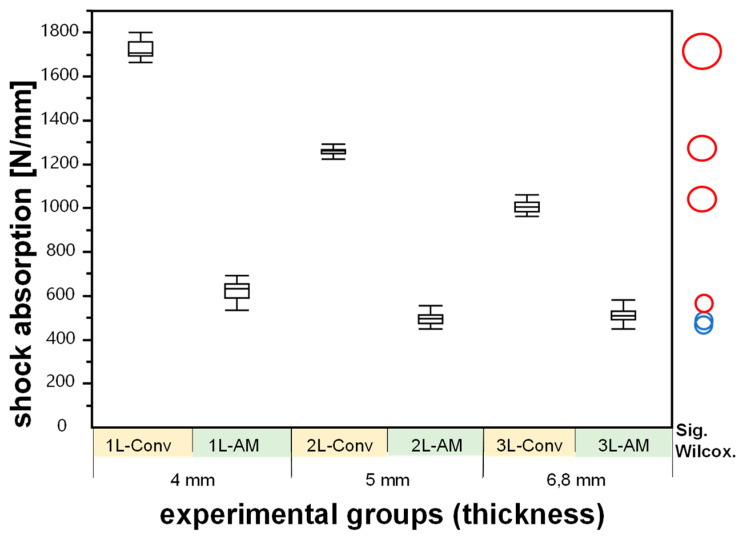
Boxplot diagram of the shock absorption per mm (y-axis) in the experimental groups (N = 35 and x-axis). The comparison circles plot on the right side indicates the confidence interval by the circles’ diameter and the statistical significance between two groups (Wilcoxon multiple comparison, *p* < 0.05) if the angle of intersection of the corresponding circles is fewer than 90° (red). An intersection angle of more than 90° indicates statistical insignificance (blue).

The mean maximum measured resulting force during the steel ball’s impact onto the polymer specimen and the thickness-dependent shock absorption are shown in Table 3, Figure 2 and Figure 3. All measurements were detected as normally distributed, except for 1L-AM-4 (*p* = 0.033).

The reference group No-MG had the highest mean in maximum measured force, and the conventionally manufactured group 1L-Conv-4 the lowest. The groups 1L-AM-4 and 2L-AM-5 were not statistically significant, all other groups differed statistically significantly (cf. Figure 2)

**Figure 4:**
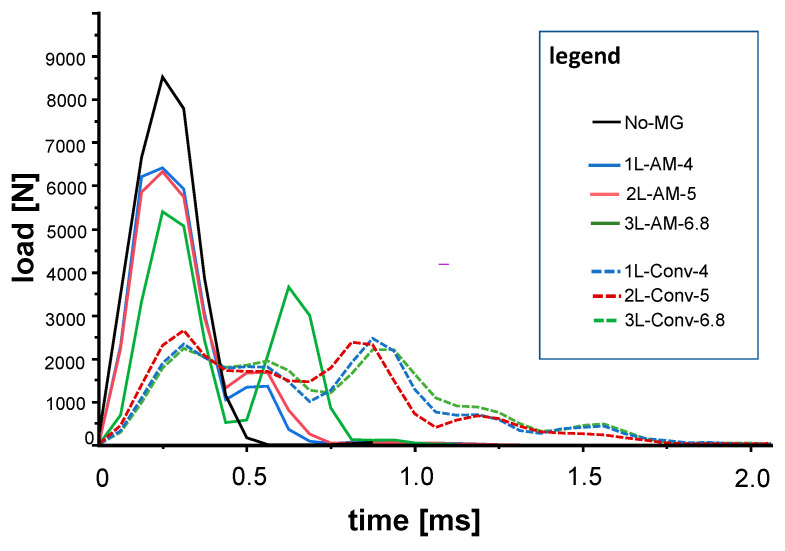
Load registration during 2 milliseconds of impact for the reference (black line), AM-group (solid lines), and conventional group (dashed lines). The integral underneath each curve is the momentum transfer (in Ns, see Table 3).

The force measurements of the impact during two milliseconds of recording is shown in Figure 4. All groups except No-MG showed more than one peak in the force recording. For the AM group, the maximum force is reached with the first recorded peak; for the conventional groups, both peaks reveal almost the same height. No-MG has the shortest impact time from zero to maximum force and back to zero.

The calculations for the ball’s momentum $\vec{p_{ball}}\;$ by the Equations (1) and (3) resulted in 1.14 Ns. The mean momentum transfer $d\vec{p}$ with Equation (6) for each group are shown in Table 3; even if these mean values were statistically significantly different for all groups, they lay between 1.77 Ns and 1.99 Ns. The summation in the Equations (4) and (5) with the unknown velocity $\vec{v_{ball}^{\prime }}$ of the ball jumping back upwards after the impact explains why the momentum transfer is an amount higher than the ball’s momentum.

More extensive data from the experiments can be found in the Supplementary Files.

## 4. Discussion

**Figure 5:**
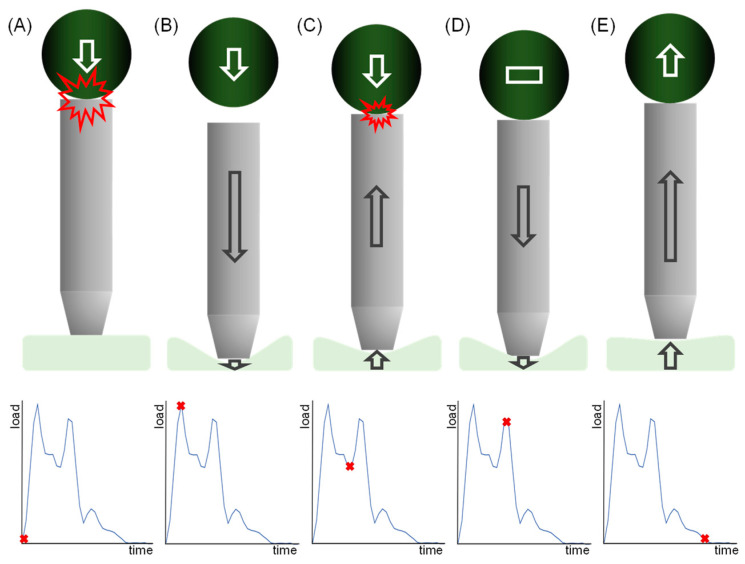
Explanatory hypothesis for the existence of two or more peaks within the test results (plots). The force from the ball’s impact is transferred over the bolt to the material sample (**A**) and recorded by the load cells during compression (**B**). The decreasing force can be explained by the reset of the material, and therewith the bolt moves upward encountering the falling ball (**C**) causing a further load impact to the bolt, material, and load cells (**D**). As no further impact happens, the bolt’s dead load is the initial baseline of the load cells (**E**).

All installed elements were necessary in order to ensure the absolutely vertical force transfer to the polymer specimen. Nevertheless, the experimental setup and the inconsistent material samples leave some room for discussion. The undamped transfer of kinetic energy, for example, which was also assumed for the theoretical calculation, is limited in reality by friction and deformation. The conventional specimen showed higher deviations from the targeted thickness and irregularly distributed air bubbles; the printed specimen seemed to be homogeneous. These properties of the conventional samples seem to be related to the analog manufacturing process (manual thermoforming), while the additive ones are fully computer controlled. Air bubbles derive from the compressed air applied to the heated resin for forming. They are not necessarily a disadvantage, as they potentially can influence the damping effect positively, but they cannot be created intentionally and can affect hygiene. In consequence, these manufacturing-related influences must be considered in order to convey the routine lab fabrication to the in vitro trial. As pointed out in the literature, in vitro models are widely limited to reconstruct forces that act on the human tooth during a sports accident [16]. It is therefore equally difficult to determine the force-dampening effect of a sports mouthguard. With the force-over-time recording during the impact (cf. Figure 5), it could be proven that the ball-drop experiment followed the physical laws summarized in Equations (1) and (2). If a polymer’s mechanical behavior delivers a spread of the temporal and spatial distribution resulting in a more continuous momentum transfer, the maximum acting force is significantly reduced. This very effect increases the mouthguards effectiveness in protecting the teeth from punctual impact of a force maximum during sports.

However, the results of this study are not to be understood as absolute numbers and any comparison of the absolute values with other studies must be refrained from. Starting from the assumed underlying physics, the examined ball-drop experiment in this study only captures the shock-absorbing effect by recording force and time but not the spatial distribution of the forces. Chowdhury et al. suggest a pressure foil (Fujifilm Prescale) in order to evaluate the distribution [16]. This could easily be included into the ball-drop experiment, too. It is assumed that an evenly distributed force over a larger area results in better shock-absorption properties.

The reference data of No-MG (∼9 kN) showed that teeth and surrounding tissue would have to absorb a much higher maximum force than with any protective layer. Absolute values may not be comparable, but 4 mm of Playsafe light material lowered the relative shock absorption to almost the same value in this study (1L-Conv-4: 71.68% of No-MG), as in Chowdhury et al. (75.93%).

Considering the force-dampening effect relative to the material thickness, the 4 mm samples showed the highest absorption to thickness ratio in both groups, conventional and AM. This 4 mm thickness is favored because a thicker mouthguard design can disturb the team communication, crucially influencing a match result [10].

The longer the force distribution time, the lower the maximum force during the impact. All material samples showing more than one peak, respectively, one peak for No-MG, can be explained by the rebounding effect of the material samples.

Depending on the material sample, this process is repeated only one or more times. All this happens within fractions of a second and in such a small scale of movement that it is not visible to the bare eye.

The difference between the theoretically calculated ball’s momentum $\vec{p_{ball}}$ and the measured and calculated mean momentum transfer $d\vec{p}$ represents the jumping back ball $\vec{p_{ball}^{\prime }}$. In the end, this momentum cannot be determined (with absolute certainty), as the entire system is also losing energy from deformation and heat development, which cannot be calculated due to too many unknown variables.

The additive-manufactured samples offer a force dampening effect in comparison with a missing protective layer. Nevertheless, the investigated additive materials do not manage to fulfill the same properties as the conventional gold-standard ones. Both the maximum shock absorption and the spreading over time is lower. Therefore, more technical development is needed in order to use additive technologies for sports mouthguard manufacturing.

However, additive manufacturing in combination with the digital design of mouth guards provides more accuracy and uniformity in manufacturing processes and the final product, as the comparison of the manufactured specimens against the intended material thicknesses with the lower standard deviations showed. With mouthguards that are additively manufactured, it may be assumed that there is no problem with thinned-out areas on cutting edges, which is the main issue of conventional thermoforming in dentistry [14]. Furthermore, since frequent use also leads to thinning, an additive-manufactured mouthguard can be replaced several times during one season without the athlete having to be present [19].

In the end, the digital workflow provides more dedicated ways to achieve force dampening. With multi-material printing, different degrees of hardness can be realized at very specific locations within the object. Advantageous and therefore designed air inclusions can be integrated using additive manufacturing, as well as cross-bracing that provides a more structural shock absorption [20].

## 5. Conclusions

Up to now, a rubberlike additively processed polymer does not appear to provide the same load-dampening effect as conventional thermoformed materials. In the future, however, AM offers the possibility of producing an optimized and individualized mouthguard. The test setup and its evaluation presented here provide a standardized method to test the emerging technologies and associated materials for their suitability. 

It was shown that not only the maximum impacting force, but also the shock-absorbing behavior in terms of temporal load distribution, needs to be investigated. Ahead of clinical approval, the mechanical dampening effect of novel materials should preferably be investigated using a simple experimental setup, such as the ball-drop method, to easily and timely ensure a direct comparison as well as comparability with other studies.

## Figures and Tables

**Table 1:** Materials and composition of the specimens made from conventional ethylenvinylacetate blanks (conventional group) and the layer thickness as well as the shore A and D hardness of the layers according to manufacturer information.

Conventional Group	1L-Conv-4	2L-Conv-5	3L-Conv-6.8
No. of specimens	N = 7	N = 7	N = 7
Manufacturer, City, and Country	Erkodent, Pfalzgrafenweiler, Germany	Scheu Dental, Iserlohn, Germany	Erkodent, Pfalzgrafenweiler, Germany
Product(Material)	Playsafe Light(ethylenvinylacetate)	Bioplast Xtreme (ethylenvinylacetate)	Playsafe Heavy Pro(ethylenvinylacetate, + styrolbutadienstyrol)
Thickness	4 mm	5 mm	6.8 mm
Composition	2 identical layers	2 layers	3 layers
Layer 1 (top)	2 mm—Shore A82	2 mm—Shore A85	2 mm—Shore A82
Layer 2	2 mm—Shore A82	3 mm—Shore A92	0.8 mm—Shore D72
Layer 3 (bottom)	none	none	4 mm—Shore A82
Thermoplastic former	ERKOFORM-RVE	BIOSTAR	ERKOFORM-RVE
Heating time (H), cooling time (C), and processing temperature	H 85 s, C 180 s, 130 °C per layer	H 140 s, C 300 s, 220 °C both layers at the same time	Layer 1: H 85 s, C 180 s, 130 °C Layer 2: H 40 s, C 45 s, 160 °C Layer 3: H 185 s, C 420 s, 120 °C

**Table 2:** Materials and composition of the specimens made from AM polymer (AM group) and the layer thickness as well as the shore A and D hardness of the layers according to terms of order and manufacturer information.

AM Group	1L-AM-4	2L-AM-5	3L-AM-6.8
No. of specimens	N = 7	N = 7	N = 7
Manufacturer, City, and Country	Stratasys, Rechovot, Israel	Stratasys, Rechovot, Israel	Stratasys, Rechovot, Israel
Product (Material)	Agilus30 (2-[[(butylamino)carbonyl]oxy]ethylacrylate) and VeroClear (Exo-1,7,7-trimethylbicyclo [2.2.1]hept-2-yl acrylate, 2-hydroxy-3-phenoxypropylacrylate)		
Thickness	4 mm	5 mm	6.8 mm
Composition:	1 layer	2 layers	3 layers
Layer 1 (top)	4 mm—Shore A80	2 mm—Shore A80	2 mm—Shore A80
Layer 2	none	3 mm—Shore A95	0.8 mm—Shore D70
Layer 3 (bottom)	none	none	4 mm—Shore A80

**Table 3:** Mean maximum forces during impact, the calculated thickness dependent force, and the calculated momentum transfer (in Ns) based on N experiments per group (No-MG = none; 1L-Conv-4, 2L-Conv-5, and 3L-Conv-6.8 = conventional; 1L-AM-4, 2L-AM-5, and 3L-AM-6.8 = additive).

Group	Experimental Group	N	Mean Maximum Force [N], (SD)	Mean Thickness-Dependent Force [N/mm], (SD)	Mean Momentum Transfer [Ns], (SD)
Ref.	No-MG	5	8982.35 (305.18)	n.a.	1.99 (0.03)
Conventional group	1L-Conv-4	35	2543.67 (95.77)	1722.45 (38.29)	1.87 (0.01)
	2L-Conv-5	35	2705.22 (84.56)	1257.41 (17.08)	1.86 (0.01)
	3L-Conv-6.8	35	2470.60 (87.00)	1006.81 (24.58)	1.95 (0.04)
AM group	1L-AM-4	35	6553.86 (168.57)	624.37 (43.28)	1.77 (0.02)
	2L-AM-5	35	6525.97 (155.41)	498.71 (31.62)	1.81 (0.03)
	3L-AM-6.8	35	5585.09 (203.99)	509.64 (30.76)	1.73 (0.03)

## Data Availability

Data is available in Supplementary Files. Raw data are available by the corresponding author upon reasonable request.

## Supplementary Materials

The following supporting information can be downloaded at: https://www.mdpi.com/article/10.3390/ma15082928/s1, Supplementary Tables in PDF File.
