# Supplementary material for: Shock Absorption Behavior of Elastic Polymers for Sports Mouthguards: An In Vitro Comparison of Thermoplastic Forming and Additive Manufacturing
Source: Materials (Basel). 2022 Apr 17;15(8):2928. doi: 10.3390/ma15082928 (PMC9028363; doi:10.3390/ma15082928)
Supplement: Supplementary file 1 [file materials-15-02928-s001.zip › materials-1600438-supplementary.pdf]

Schewe et al. 2022 * Supplementary Tables

Maximum Force (N) / Test on normal distribution

Group	n	Mean (N)	Std.dev. (N)	Shapiro-Wilk-W-Test
No-MG	5	8982,35	305,18	0,8456
1L-Conv-4	35	2543,67	95,77	0,1064
1L-AM-4	35	6553,86	168,57	0,0331
2L-Conv-5	35	2705,22	84,56	0,3950
2L-AM-5	35	6525,97	155,41	0,4909
3L-Conv-6.8	35	2470,60	87,00	0,3710
3L-Conv-6.8	35	5585,09	203,99	0,6656

Wilcoxon-Each-Pair Comparison of Maximum Force distributions

Group	- Group	Score mean difference	Std Err Dif	Z	p-Value	Hodges-Lehmann	Lower CI	Upper CL
1L-AM-4	1L-Conv-4	34,9714	4,864840	7,18861	<,0001*	3977,56	3919,98	4069,59
2L-AM-5	1L-Conv-4	34,9714	4,864840	7,18861	<,0001*	3992,63	3923,83	4050,98
2L-AM-5	2L-Conv-5	34,9714	4,864840	7,18861	<,0001*	3828,37	3765,53	3888,07
3L-AM-6.8	1L-Conv-4	34,9714	4,864840	7,18861	<,0001*	3048,96	2976,33	3120,36
3L-AM-6.8	2L-Conv-5	34,9714	4,864840	7,18861	<,0001*	2885,89	2824,48	2949,27
3L-AM-6.8	3L-Conv-6.8	34,9714	4,864840	7,18861	<,0001*	3126,38	3052,91	3189,48
2L-Conv-5	1L-Conv-4	28,1143	4,864840	5,77908	<,0001*	156,97	113,86	205,44
No-MG	1L-Conv-4	19,8857	5,589105	3,55794	0,0004*	6416,65	6214,49	6668,76
No-MG	1L-AM-4	19,8857	5,589105	3,55794	0,0004*	2402,01	2168,79	2647,86
No-MG	2L-Conv-5	19,8857	5,589105	3,55794	0,0004*	6221,18	6053,72	6473,88
No-MG	2L-AM-5	19,8857	5,589105	3,55794	0,0004*	2409,04	2190,53	2698,88
No-MG	3L-Conv-6.8	19,8857	5,589105	3,55794	0,0004*	6461,55	6291,93	6744,42
No-MG	3L-AM-6.8	19,8857	5,589105	3,55794	0,0004*	3366,32	3109,46	3676,99
2L-AM-5	1L-AM-4	-0,5714	4,864840	-0,11746	0,9065	-7,14	-91,94	71,26
3L-Conv-6.8	1L-Conv-4	-14,6857	4,864840	-3,01875	0,0025*	-79,26	-117,15	-30,77
3L-Conv-6.8	2L-Conv-5	-32,8000	4,864840	-6,74226	<,0001*	-235,92	-282,55	-197,12
2L-Conv-5	1L-AM-4	-34,9714	4,864840	-7,18861	<,0001*	-3816,29	-3884,30	-3762,63
3L-Conv-6.8	1L-AM-4	-34,9714	4,864840	-7,18861	<,0001*	-4053,85	-4128,98	-3994,85
3L-Conv-6.8	2L-AM-5	-34,9714	4,864840	-7,18861	<,0001*	-4062,02	-4124,00	-4000,61
3L-AM-6.8	1L-AM-4	-34,9714	4,864840	-7,18861	<,0001*	-943,10	-1044,94	-861,31
3L-AM-6.8	2L-AM-5	-34,9714	4,864840	-7,18861	<,0001*	-938,98	-1021,69	-857,14

Schewe et al. 2022 * Supplementary Tables

Shockabsorption per mm (N/mm) / Test on normal distribution

Group	n	Mean (N/mm)	Std.dev. (N/mm)	Shapiro-Wilk-W-Test
1L-Conv-4	35	1722,45	38,29	0,0255
1L-AM-4	35	624,37	43,28	0,0290
2L-Conv-5	35	1257,41	17,08	0,4605
2L-AM-5	35	498,71	31,62	0,5094
3L-Conv-6.8	35	1006,81	24,58	0,6104
3L-AM-6.8	35	509,64	30,76	0,6478

Wilcoxon-Each-Pair Comparison of Schockabsorption per mm distributions

Group	- Group	Score mean difference	Std Err Dif	Z	p-Value	Hodges-Lehmann	Lower CI	Upper CL
2L-Conv-5	1L-AM-4	34,9714	4,864840	7,18861	<,0001*	625,68	611,88	639,71
3L-Conv-6.8	1L-AM-4	34,9714	4,864840	7,18861	<,0001*	375,21	359,90	394,39
3L-Conv-6.8	2L-AM-5	34,9714	4,864840	7,18861	<,0001*	509,57	494,73	523,40
3L-AM-6.8	2L-AM-5	7,2000	4,864840	1,48001	0,1389	11,41	-3,08	26,39
3L-AM-6.8	1L-AM-4	-33,3714	4,864840	-6,85972	<,0001*	-121,76	-137,71	-101,54
2L-AM-5	1L-AM-4	-34,0000	4,864840	-6,98892	<,0001*	-131,45	-150,04	-111,97
1L-AM-4	1L-Conv-4	-34,9714	4,864840	-7,18861	<,0001*	-1095,01	-1117,23	-1070,21
2L-Conv-5	1L-Conv-4	-34,9714	4,864840	-7,18861	<,0001*	-458,71	-477,71	-443,46
2L-AM-5	1L-Conv-4	-34,9714	4,864840	-7,18861	<,0001*	-1221,29	-1241,15	-1203,13
2L-AM-5	2L-Conv-5	-34,9714	4,864840	-7,18861	<,0001*	-760,05	-772,52	-748,47
3L-Conv-6.8	1L-Conv-4	-34,9714	4,864840	-7,18861	<,0001*	-711,21	-730,00	-695,60
3L-Conv-6.8	2L-Conv-5	-34,9714	4,864840	-7,18861	<,0001*	-252,43	-261,49	-241,03
3L-AM-6.8	1L-Conv-4	-34,9714	4,864840	-7,18861	<,0001*	-1208,61	-1228,96	-1191,65
3L-AM-6.8	2L-Conv-5	-34,9714	4,864840	-7,18861	<,0001*	-749,73	-759,41	-738,54
3L-AM-6.8	3L-Conv-6.8	-34,9714	4,864840	-7,18861	<,0001*	-497,10	-510,91	-484,30

Schewe et al. 2022 * Supplementary Tables

Momentum Transfer (Ns) / Test on normal distribution

Group	n	Mean (Ns)	Std.dev. (Ns)	Shapiro-Wilk-W-Test
No-MG	5	1,9867616	0,031619	0,3853
1L-Conv-4	35	1,8697638	0,0141259	0,8480
1L-AM-4	35	1,7736708	0,0173167	0,0074
2L-Conv-5	35	1,8603856	0,0131148	0,0051
2L-AM-5	35	1,8141323	0,0279953	0,0008
3L-Conv-6.8	35	1,9456692	0,0424072	<0,0001
3L-Conv-6.8	35	1,7309591	0,0346721	0,1847

Wilcoxon-Each-Pair Comparison of Momentum Transfer distributions

Group	- Group	Score mean difference	Std Err Dif	Z	p-Value	Hodges Lehmann	Lower CI	Upper CL
2L-Conv-5	1L-AM-4	34,9714	4,864840	7,18861	<,0001*	0,085400	0,078070	0,091776
3L-Conv-6.8	1L-AM-4	34,9714	4,864840	7,18861	<,0001*	0,177978	0,169584	0,188899
3L-Conv-6.8	2L-AM-5	32,2286	4,864840	6,62480	<,0001*	0,137972	0,126605	0,146382
2L-AM-5	1L-AM-4	29,6000	4,864840	6,08448	<,0001*	0,043391	0,032085	0,051538
3L-Conv-6.8	2L-Conv-5	29,1429	4,864840	5,99051	<,0001*	0,092616	0,085628	0,100554
3L-Conv-6.8	1L-Conv-4	29,0286	4,864840	5,96701	<,0001*	0,085009	0,075483	0,093104
NO-MG	1L-Conv-4	19,8857	5,589105	3,55794	0,0004*	0,123269	0,097544	0,141839
NO-MG	1L-AM-4	19,8857	5,589105	3,55794	0,0004*	0,219278	0,193859	0,236564
NO-MG	2L-Conv-5	19,8857	5,589105	3,55794	0,0004*	0,130724	0,108121	0,151361
NO-MG	2L-AM-5	19,8857	5,589105	3,55794	0,0004*	0,174920	0,144568	0,198917
NO-MG	3L-AM-6.8	19,8857	5,589105	3,55794	0,0004*	0,259481	0,222377	0,287497
NO-MG	3L-Conv-6.8	12,3429	5,589105	2,20838	0,0272*	0,037406	0,006903	0,061467
2L-Conv-5	1L-Conv-4	-12,5143	4,864840	-2,57239	0,0101*	-0,008501	-0,015772	-0,001936
3L-AM-6.8	1L-AM-4	-25,3714	4,864840	-5,21526	<,0001*	-0,043917	-0,054931	-0,030687
2L-AM-5	2L-Conv-5	-32,1143	4,864840	-6,60130	<,0001*	-0,043955	-0,052817	-0,034540
3L-AM-6.8	2L-AM-5	-32,5714	4,864840	-6,69527	<,0001*	-0,085913	-0,099122	-0,071365
2L-AM-5	1L-Conv-4	-34,1714	4,864840	-7,02416	<,0001*	-0,052597	-0,061139	-0,044066
1L-AM-4	1L-Conv-4	-34,9714	4,864840	-7,18861	<,0001*	-0,094507	-0,101683	-0,087829
3L-AM-6.8	1L-Conv-4	-34,9714	4,864840	-7,18861	<,0001*	-0,139788	-0,150684	-0,126918
3L-AM-6.8	2L-Conv-5	-34,9714	4,864840	-7,18861	<,0001*	-0,129737	-0,141454	-0,118846
3L-AM-6.8	3L-Conv-6.8	-34,9714	4,864840	-7,18861	<,0001*	-0,221583	-0,236040	-0,208335

Schewe et al. 2022 * Supplementary Tables

Specimen Thickness (mm) / Test on normal distribution

Group	n	Mean (mm)	Std.dev. (mm)	Shapiro-Wilk-W-Test
1L-Conv-4	35	3,74	0,091	0,0134
1L-AM-4	35	3,889	0,004	0,0003
2L-Conv-5	35	4,992	0,043	0,0080
2L-AM-5	35	4,925	0,003	<0,0001
3L-Conv-6.8	35	6,47	0,132	0,0059
3L-Conv-6.8	35	6,666	0,005	0,0039

Wilcoxon-Each-Pair Comparison of thickness distributions

Group	- Group	Score mean difference	Std Err Dif	Z	p-Value	Hodges-Lehmann	Lower CI	Upper CL
1L-AM-4	1L-Conv-4	34,9714	4,852909	7,20628	<,0001*	0,13040	0,10180	0,18400
2L-Conv-5	1L-Conv-4	34,9714	4,852909	7,20628	<,0001*	1,24440	1,21020	1,29300
2L-Conv-5	1L-AM-4	34,9714	4,852909	7,20628	<,0001*	1,10900	1,07980	1,11580
2L-AM-5	1L-Conv-4	34,9714	4,847573	7,21421	<,0001*	1,16400	1,13540	1,21760
2L-AM-5	1L-AM-4	34,9714	4,847573	7,21421	<,0001*	1,03480	1,03340	1,03900
3L-Conv-6.8	1L-Conv-4	34,9714	4,852909	7,20628	<,0001*	2,73200	2,67940	2,79320
3L-Conv-6.8	1L-AM-4	34,9714	4,852909	7,20628	<,0001*	2,59880	2,49540	2,66260
3L-Conv-6.8	2L-Conv-5	34,9714	4,852909	7,20628	<,0001*	1,48980	1,41560	1,54720
3L-Conv-6.8	2L-AM-5	34,9714	4,847573	7,21421	<,0001*	1,56520	1,46180	1,62780
3L-AM-6.8	1L-Conv-4	34,9714	4,852909	7,20628	<,0001*	2,90800	2,87940	2,96160
3L-AM-6.8	1L-AM-4	34,9714	4,852909	7,20628	<,0001*	2,77740	2,77440	2,77900
3L-AM-6.8	2L-Conv-5	34,9714	4,852909	7,20628	<,0001*	1,66860	1,65900	1,69780
3L-AM-6.8	2L-AM-5	34,9714	4,847573	7,21421	<,0001*	1,74140	1,73860	1,74440
3L-AM-6.8	3L-Conv-6.8	24,9714	4,852909	5,14566	<,0001*	0,17880	0,11560	0,28220
2L-AM-5	2L-Conv-5	-32,1143	4,847573	-6,62482	<,0001*	-0,07540	-0,08200	-0,04620
